# Zero violence or zero seclusion. Which is more acceptable in our hospitals?

**DOI:** 10.1192/j.eurpsy.2022.1539

**Published:** 2022-09-01

**Authors:** K. Tong, A. Gibbons, O. Byrne, T. Conlon, H. Kennedy, M. Davoren

**Affiliations:** 1 National Forensic Mental Health Service, Central Mental Hospital, Dundrum, Dublin, Ireland; 2 Central Mental Hospital and Trinity College Dublin, Dundrum Centre For Forensic Excellence, Dublin, Ireland

**Keywords:** violence, Restrictive practice, seclusion, mental illness

## Abstract

**Introduction:**

There is an established association between serious mental illness and violence. Secure forensic psychiatric services provide care and treatment to mentally disordered offenders. The majority of patients in forensic services suffer from severe mental illnesses such as schizophrenia, with co-morbid polysubstance abuse and maladaptive personality traits. Psychiatric services are under significant pressure to reduce the use of seclusion and restrictive practices, whilst mandated to provide safe environments for patients and staff.

**Objectives:**

To determine the number and characteristics of violent incidents in a secure forensic hospital in Ireland.

**Methods:**

A retrospective review of all incidents in Central Mental Hospital, Ireland between 1^st^ March 2019 and 31^st^ August 2021 was completed. Incidents were categorised into physical assaults and other violent incidents. Demographic measures and measures of violence risk (HCR-20), functioning (GAF), programme completion and recovery (DUNDRUM tool) were collated.

**Results:**

A total of 321 incidents took place during the period examined, of which 47 (14.6%) involved physical assaults perpetrated by patients. Between March 2020 and August 2021, numbers of assaults increased by 50% and 78% compared to the preceding six-month period respectively. The majority of assaults were committed by a relatively small group of patients. Victims of assaults were more likely to be patients (n=27, 57.4%) and more likely to be males (n=43, 91.9%).

**Conclusions:**

Physical assaults and other violent incidents happen in forensic and general psychiatric units. Restrictive practices, used in accordance with the law, are necessary at times to prevent serious harm to patients and staff in psychiatric hospitals.

**Disclosure:**

No significant relationships.

